# Multi-ancestry GWAS of Fuchs corneal dystrophy highlights roles of laminins, collagen, and endothelial cell regulation

**DOI:** 10.21203/rs.3.rs-2762003/v1

**Published:** 2023-05-03

**Authors:** Neal Peachey, Bryan Gorman, Michael Francis, Cari Nealon, Christopher Halladay, Nalvi Duro, Kyriacos Markianos, Giulio Genovese, Pirro Hysi, Hélène Choquet, Natalie Afshari, Yi-Ju Li, J. Michael Gaziano, Adriana Hung, Wen-Chih Wu, Paul Greenberg, Saiju Pyarajan, Jonathan Lass, Sudha Iyengar

**Affiliations:** Louis Stokes Cleveland VA; VA Boston Healthcare System; Booz Allen Hamilton; VA Northeast Ohio Healthcare System; Providence VA Medical Center; VA Boston Healthcare System; VA Boston Healthcare System; Broad Institute; King's College London; Division of Research, Kaiser Permanente Northern California; UC San Diego, Shiley Eye Institute; Duke University; VA Boston Healthcare System; Vanderbilt University Medical Center; Providence VA Medical Center; Providence VA Medical Center; Center for Data and Computational Sciences, Veterans Affairs Boston Healthcare System; Case Western Reserve University and University Hospitals Case Medical Center; Case Western Reserve University

## Abstract

Fuchs endothelial corneal dystrophy (FECD) is a leading indication for corneal transplantation, but its molecular pathophysiology remains poorly understood. We performed genome-wide association studies (GWAS) of FECD in the Million Veteran Program (MVP) and meta-analyzed with the previous largest FECD GWAS, finding twelve significant loci (eight novel). We further confirmed the TCF4 locus in admixed African and Hispanic/Latino ancestries, and found an enrichment of European-ancestry haplotypes at TCF4 in FECD cases. Among the novel associations are low frequency missense variants in laminin genes LAMA5 and LAMB1 which, together with previously reported LAMC1, form laminin-511 (LM511). AlphaFold 2 protein modeling suggests that mutations at LAMA5 and LAMB1 may destabilize LM511 by altering inter-domain interactions or extracellular matrix binding. Finally, phenome-wide association scans and co-localization analyses suggest that the TCF4 CTG18.1 trinucleotide repeat expansion leads to dysregulation of ion transport in the corneal endothelium and has pleiotropic effects on renal function.

## Introduction

Fuchs endothelial corneal dystrophy (FECD) is the most common corneal dystrophy, affecting more than 5% of people older than 40 years of age, and is the leading indication for corneal transplantation (keratoplasty) in the United States^1^. Globally, only 1 in 70 people needing a corneal transplant receive one^2^, and a portion of transplants result in graft rejection or failure^3^, demonstrating the need for early prediction of genetic susceptibility, as well as pharmacologic alternatives, for treating FECD.

FECD is a progressive, bilateral disease^4,5^. Earliest indications of FECD are the presence of excreted collagenous deposits called guttae^6-8^. As FECD progresses, guttae grow in numbers and merge, leading to a thickening of Descemet’s membrane. These changes put stress on corneal endothelial cells (CECs), which regulate solute transfer and flow of water into the stroma. CECs begin to undergo cell death via apoptosis^9,10^, accompanied by measurable changes in CEC shape and density^11^, corneal biomechanics^12,13^, and central corneal thickness (CCT)^14,15^. Disruption of endothelium function leads to corneal edema, resulting in blurred vision and eventual severe vision loss.

The etiology of FECD involves complex interactions between incompletely penetrant genetic factors and biological and environmental factors^16^. Female sex and advanced age are established risk factors. Risk may also differ across populations; lower rates of FECD diagnosis have been observed in African Americans in both clinical settings^17^ and Medicare claims^18^. Similarly, examining FECD by genetic ancestry in the Department of Veterans Affairs Million Veteran Program (MVP), we found significantly reduced prevalence in participants of admixed African (AFR) and Hispanic/Latino (HIS) continental ancestries relative to European ancestry (EUR)^19^.

The first genetic risk factors for FECD were discovered in family studies^20^ and included ultra-rare mutations in *COL8A2*^21^ and *SLC4A11*^22^. Subsequently, genome-wide association studies (GWAS) identified four risk loci for FECD. Of these, the most significant was common variation at 18q21.2^23^ tagging the CTG18.1 trinucleotide repeat (TNR) expansion in an intron of *TCF4*^24^ (transcription factor 4). As many as 75% of European-ancestry FECD cases had at least one expanded CTG18.1 allele^25^. The previous largest FECD GWAS to date^26^ confirmed *TCF4* and identified three additional loci: *LAMC1, KANK4,* and *ATP1B1*.

Recent genetic studies of FECD in non-European ancestries have largely focused on the genotyping and association of CTG18.1 alleles. CTG18.1 expansions are associated with FECD in African American^17^, Chinese^27^, Indian^28^, Japanese^29^, and Thai^30^ populations. However, CTG18.1 expansions are generally observed at relatively lower frequencies in non-European ancestry FECD patients compared to European-descent FECD patients^17,31^. It remains unclear whether the population frequency of penetrant CTG18.1 alleles differs by genetic ancestry.

Here, we leverage genetic and clinical data provided by the MVP to conduct the largest GWAS analysis of FECD and first multi-ancestry GWAS meta-analysis. We confirm the four previously reported loci, including the presence of the *TCF4* GWAS locus in AFR and HIS, and present eight novel loci, expanding our knowledge of the genetic drivers of FECD.

## Results

### Multi-ancestry GWAS for FECD

**Figure 1:**
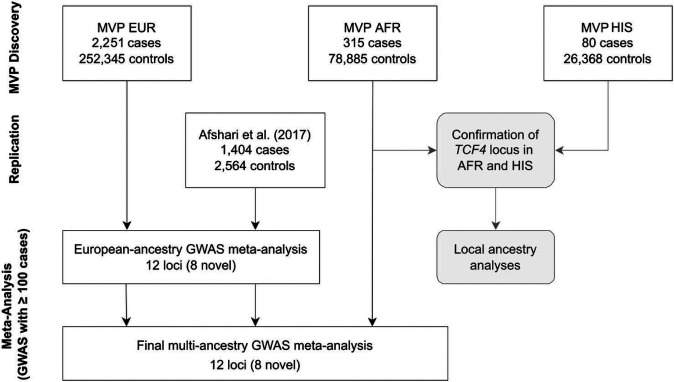
GWAS overview. Discovery analyses were performed in MVP European (EUR), admixed African (AFR), and Hispanic/Latino (HIS) cohorts. Numbers of FECD cases and controls are shown. Local ancestry analyses were performed in AFR and HIS cohorts. HIS were not included in multi-ancestry meta-analysis due to the low number of FECD cases. Afshari et al.^26^ was included as a replication cohort.

We identified FECD cases in MVP participants of EUR, AFR, and HIS ancestry (Supplementary Table 1) following a clinically validated phenotyping algorithm^19^. Cases were mostly male (88.6%), reflecting the predominantly male composition of the MVP dataset^32^. As FECD is more common in women, there was a higher frequency of female cases vs. controls in each ancestry group (combined 11.4% cases vs. 8.4% controls). Mean age of FECD cases ranged from 62.8 [95% Confidence Interval (CI)=52.7, 72.9] in AFR to 70.5 [60.9, 80.1] years old in EUR. We performed a mixed-model GWAS for FECD using SAIGE^33^ for each ancestry (Fig. 1, Supplementary Fig. 1), including age, age-squared, sex, and ten ancestry-specific principal components as covariates.

The *TCF4* locus reached genome-wide significance (GWS; *P*<5×10 ^8^) across all three ancestries analyzed in MVP; this was the first time *TCF4* has been significantly associated with FECD in a GWAS in AFR or HIS (Table 1; Supplementary Fig. 1a-c). The lead SNP at *TCF4,* rs11659764 (r^2^=0.21 and D’=0.97 with the previously reported FECD index variant, rs613872), was the same across all three ancestries. Although the marker varied in frequency, the additive effect of each allele of rs11659764 on FECD was highly similar across ancestries, and we concur that the lower frequency of CTG18.1 TNR expansions observed in cases in AFR-descent populations contributes to a reduction in FECD risk^17^.

We applied local ancestry admixture mapping models at the *TCF4* locus in AFR and HIS to directly compare risk conferred by haplotype ancestry within the same individuals. In the AFR population, each EUR haplotype was additively associated with FECD (odds ratio (OR) = 1.28 [1.02, 1.61] (*P*=0.015), with 23% frequency of EUR haplotypes in cases vs. 18% in controls. In HIS, we found a similar OR for EUR haplotypes relative to AFR and Native American ancestry (NAT) haplotypes (OR=1.27 [0.91, 1.78]; *P*=0.17), with 64% EUR haplotype frequency in cases vs. 57% in controls, but this was non-significant due to lower power. Consistent with allele frequencies at our lead tagging SNP rs11659764, this result suggests that EUR haplotypes contain a higher frequency of pathogenic alleles compared to AFR and possibly also NAT haplotypes. The sample sizes for the MVP Asian cohorts were too low to obtain reliable estimates, but data from prior studies in Japanese cohorts and allele frequencies from the 1000 Genomes Project suggest that East Asians also have lower frequency of CTG18.1 expansions, contributing to a lower FECD risk^34^.

Seven loci were GWS in the MVP EUR discovery cohort, including all four known FECD GWAS loci^26^ (*TCF4, KANK4, LAMC1,* and *ATP1B1*) and three novel loci at *SSBP3, THSD7A,* and *PIDD1* (Supplementary Table 2; Supplementary Fig. 1a). Previously, a SNP at the *PIDD1* gene locus was reported to have reached suggestive significance^26^ (*P*=7×10^−7^), while our lead novel variants at *SSBP3* and *THSD7A* were at least nominally significant (*P*=2.61×10^−5^ and *P*=0.025, respectively).

We then performed inverse variance-weighted fixed effects meta-analyses, first exclusively across the two European cohorts, MVP EUR and Afshari et al.^26^ (Supplementary Fig. 1d), and finally a multi-ancestry meta-analysis which added MVP AFR (HIS were excluded due to fewer than 100 cases; Fig. 1). This multi-ancestry meta-analysis tested a total of 18,302,074 variants in up to 3,970 cases and 333,794 controls (Supplementary Table 1), ~2.8 times the case sample size of the previous largest FECD GWAS cohort.

**Figure 2:**
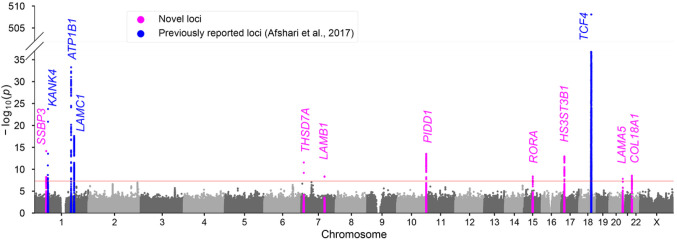
Manhattan plot of the multi-ancestry meta-analysis. Plot shows the −log10(*P*) for associations of genetic variants with FECD across 22 autosomal chromosomes plus chromosome X. Significant loci are highlighted in magenta (novel) and blue (previously reported). The red line indicates the genome-wide significance threshold (*P* < 5 × 10 ^8^).

In the multi-ancestry meta-analysis, we replicated the 4 previously reported loci and identified a total of eight novel FECD loci emerging at GWS: *LAMA5, LAMB1, COL18A1, SSBP3, THSD7A, RORA, PIDD1,* and *HS3ST3B1* (Table 2; Fig. 2; Supplementary Fig. 2). Genomic control (λ) was 1.01, indicating minimal systematic inflation. Stepwise conditional and joint association analysis (COJO-slct) of the lead variant in each locus indicated no additional independent signals reaching GWS (*TCF4* was excluded due to the untyped CTG18.1 trinucleotide repeat).

As expected, the largest OR was observed at rs11659764 in *TCF4* (OR=7.15, [6.60, 7.74]; Supplementary Fig. 3). Effect sizes at index SNPs were consistent across MVP EUR and Afshari et al^26^, and all meta-analysis index SNPs were at least nominally significant (*P*<0.05) in the prior report, further validating our phenotyping approach. Six of twelve index variants did not meet the meta-analysis minor allele frequency (MAF) cutoff of ≥1% in AFR. Additionally, all index SNPs had consistent effect direction in AFR, with the exception of rs12439253 (*RORA*), which had a non-significant and opposite effect direction (Supplementary Fig. 4), Two AFR meta-analysis SNPs, rs1138714 in *PIDD1* and rs114065856 in *COL18A1,* had consistent direction with EUR cohorts but were not significant.

Linkage disequilibrium score regression (LDSC) analysis indicated the total liability-scale SNP heritability (SNP-*h*^2^) for FECD, based on EUR meta-analysis summary statistics, was 0.43 (standard error [SE] = 0.32), assuming a 5% population prevalence. As LDSC generally measures polygenicity^35^, the uncertainty of the SNP-*h*^2^ estimate may reflect the partially monogenic (*TCF4*) architecture of FECD.

### Novel FECD candidate genes

We identified candidate genes for our eight novel GWAS loci in the biological context of FECD; these are summarized in Table 3. Two novel loci emerged with lead variants in laminin genes: *LAMA5* ( 5) and *LAMB1* (β1). Together with the previously reported *LAMC1* (γ1) protein, these subunits form the laminin-511 heterotrimer (LM511; also called laminin-10), implicating an important role for LM511 in CEC maintenance and FECD pathogenesis. In previous studies, LM511 staining patterns were thicker in FECD corneas than controls^36^; additionally, LM511 facilitated the expansion of CECs in culture^37^ and promoted recovery of CECs in animal models of CEC transplantation^38^. At *LAMB1,* our association peak consisted of three low-frequency (1-2% in EUR) variants in LD (r^2^>0.9; Supplementary Fig. 2), each with a posterior inclusion probability (PIP) of 30-35% estimated from sum of single effects (SuSiE) fine-mapping^39,40^. Of these, the most likely causal variant is the missense mutation at rs80095409 (p.Arg795Gly), which was computationally predicted to have a deleterious impact on protein structure by both SIFT^41^ and Polyphen^42^ classifiers, with a Combined Annotation Dependent Depletion (CADD) score of 29.7^43^ (Supplementary Fig. 5A). Interestingly, the *LAMB1* locus has no pleiotropy with other traits reported in the GWAS Catalog.

At *LAMA5,* the characteristic subunit of LM511, the lead variant rs141208202 is also a low-frequency (4-5% in EUR) missense mutation, p.Gly2156Glu, that was predicted by SIFT to be deleterious and had 78% PIP. The next most significant variant (rs143905087; *P*=6.74×10 ^8^), is an intronic variant in *CABLES2* in only moderate LD with the lead variant (r^2^=0.54; Supplementary Fig. 5B) and 17% PIP. Thus, we prioritize rs141208202 as a likely causal variant at *LAMA5,* mediated through putative impact on protein structure, which we explore further below. However, rs141208202 is a *LAMA5* splicing quantitative trait locus (QTL) in some tissues in GTEx^44^ and is located within a CTCF binding site^45^, and thus may also have a regulatory impact.

We discovered two novel loci likely driven by collagen genes: *SSBP3* and *COL18A1*. SSBP3 (single-stranded DNA binding protein 3) is predicted to bind to a polypyrimidine tract in the promoter of *COL1A2* and regulate its expression^46^. Collagen type I is one of the primary collagens found in corneal tissue, and other subunits of collagen type I have emerged in previous GWAS of corneal traits^47^. *COL18A1* encodes the alpha chain of type XVIII collagen, a ubiquitous component of the basement membrane (BM). In addition to its structural role, cleavage of type XVIII collagen generates the regulatory peptide endostatin, which inhibits proliferation of endothelial cells through G1 arrest^48^ and can induce cell death, implicating anti-tumorigenic and anti-angiogenic properties of this domain^49^.

Another novel locus was identified at *THSD7A*. THSD7A interacts with integrin alpha V beta 3 (αvβ3)^50,51^, expressed on CECs^52^, to inhibit migration. *THSD7A* has been previously associated in GWAS studies with four ocular traits: glaucoma, intraocular pressure, refractive error, and cataract (Supplementary Table 3). Though the lead variants for these associations are in *THSD7A,* they have low r^2^ with our lead SNP rs74882680 (r^2^ ≤ 0.015), due to multiple distinct LD blocks within this gene (Supplementary Fig. 2).

The lead variant of a gene-dense region at 11p15.5, rs1138714, corresponds to several potential candidate genes (Supplementary Fig. 2). *CD151*, whose gene product is a member of the tetraspanin family, is an essential global regulator of endothelial cell-cell and cell-matrix adhesion^53^. Like THSD7A, CD151 is involved in integrin binding via the formation of tetraspanin-enriched microdomains. Additionally, CD151, along with type XVIII collagen and laminins, are members of the collagen chain trimerization pathway. Another gene at 11p15.5, PNPLA (AKA desnutrin or TTS-2.2), is a paralogue with similar properties to hGS2, which is responsible for transferring fatty acids from triglycerides to retinol, as well as hydrolyzing retinylesters^54^. Adequate retinol is required for corneal development and function, and CECs are involved in the conversion of retinol into retinoic acid^55^. Finally, PIDD1 also has a potential role in regulating CEC death via apoptosis. *PNPLA2* and *PIDD1* were differentially expressed in CEC in patients with keratoconus (KC) and myopia^56^. The association of rs1138714 with eQTLs at all three of these biologically relevant genes in GTeX indicates that synchronized co-expression of multiple causal genes in this region may also be possible^44^. This locus has been previously associated with multiple ocular traits, and our FECD index variant at rs1138714 is in strong LD with rs10902223 (r^2^=0.99), reported as the lead variant for KC and intraocular pressure, and is also in moderate LD with rs4963153 (r^2^=0.54), the lead variant reported for associations with corneal resistance factor (CRF) and CCT (Supplementary Table 3).

We identified a novel association with FECD at *RORA,* which belongs to the family of retinoic acid-related orphan receptors (RORs). RORs are a superfamily of nuclear receptor transcription factors which bind to hormone response units. Although it shares structural features with retinoic acid receptors (RARs), RORA does not have known ligand-binding properties with retinol. RORA is most commonly associated with regulation of *BMAL1* and circadian rhythm; CECs have a highly robust circadian clock, and FECD and other corneal maladies are known to exhibit diurnal variation^57^. *RORA* is induced by oxidative stress; reduction of *NFE2L2* nuclear factor translocation, which leads to downregulation of antioxidant expression, has previously been observed in FECD cases^58^. RORA also regulates the differentiation and maintenance of type-2 innate lymphoid cells^59,60^, which are among the immune cells resident in the cornea^61^. Additionally, our top FECD index SNP at *RORA,* rs12439253, has r^2^=0.59 with the KC index SNP rs76194223.

Finally, a novel FECD locus was found in an intergenic region ~314 Kbp downstream from the nearest coding gene, *HS3ST3B1.* HS3ST3B1 is a 3-O-sulfotransferase integral membrane protein, which catalyzes the addition of sulfate groups to heparan sulfate (HS). HS is required for a wide range of cellular processes, including by CECs for maintaining corneal homeostasis^62^. Heparanase (HPSE), which acts as a protease of HS in the BM, was overexpressed in keratoconic corneas, and HPSE catalytic activity was correlated with KC severity^63^. In addition, a severe impediment to corneal wound healing was observed in a mouse HS knockout model^62^. This locus has been previously associated with CCT in three GWAS studies (r^2^=0.95 to 1) and CEC size variation coefficient (r^2^=0.63).

Intriguingly, a locus near *ANAPC1* previously reported to account for 24% of variability in CEC density in an Icelandic population^64^ reached suggestive levels of significance in our multi-ancestry meta-analysis. However, the allele reported to decrease CEC density (rs78658973-A) was protective for FECD (OR=0.86 [0.80, 0.92]; *P*=5.1×10 ^6^). In the same study, this allele was also significantly associated with increased coefficient of cell size variation and decreased percentage of hexagonal cells. The other allele reported to decrease CEC density, the CTG18.1 TNR expansion, greatly increases risk of FECD (Supplementary Table 3). Thus, our results support a complex relationship between CEC density and FECD, with potential bidirectional causality.

### Pleiotropy of FECD risk alleles

#### Index variant directional trends across four corneal traits

**Figure 3:**
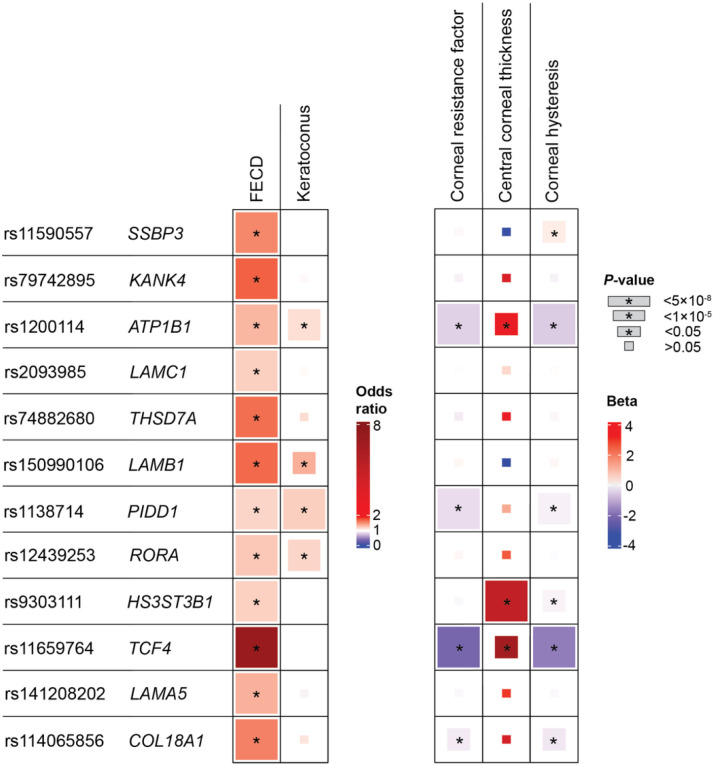
Comparing effects of FECD with four corneal traits. Variant-level comparison of lead FECD variants with four other corneal traits: keratoconus (KC), central corneal thickness (CCT), corneal resistance factor (CRF), and corneal hysteresis (CH). Box sizes correspond to *P*-value tiers, and * indicates *P* < 0.05. Units: FECD, odds ratio; KC, odds ratio; CCT, μm; CRF, mm Hg; CH, mm Hg.

We compared the effect size and direction of our lead FECD variants with summary statistics from other corneal traits: KC^47^, CCT^65,66^, CRF^67^, and corneal hysteresis (CH)^68^. We found consistent directional trends in a variant-level comparison across these traits. Eight of twelve FECD index variants had nominally significant associations (*P*<0.05) in at least one other corneal trait. At the nominally significant variants for each respective trait, all KC and CCT variant effects were in the same direction as FECD, while all CRF variants, and all variants but one in CH (*SSBP3*) were associated with effects in the opposite direction, consistent with previous reports^12^ (Fig. 3; Supplementary Table 4). Genetic correlations (*r*_*g*_) between FECD and these traits were not significant, however they followed the same directional pattern as the variant-level trends.

#### Association of PGS Catalog-based polygenic scores with FECD status

We calculated polygenic scores (PGS) for every trait in the PGS Catalog^69^, for all MVP EUR subjects, and performed a phenome-wide scan for the association of normalized PGS scores with FECD case-control status to discover shared genetic etiology (Supplementary Table 5). A total of 2,649 scores corresponding to 560 uniquely mapped traits in the Experimental Factor Ontology (EFO) were tested; we considered 24 traits to be significant after adjustment for multiple comparisons (*P*<0.05/560). We found that PGSs for other corneal traits had the strongest associations with FECD, including CH (OR=0.83 [0.79, 0.86]; *P*=7.04×10 ^20^) and CRF (OR =0.86 [0.83, 0.90]; *P*=4.73×10 ^12^). The negative effect direction of these corneal trait PGS associations is consistent with our variant-level analysis in Fig. 3. After corneal traits, several renal PGSs had significant associations with FECD status, including urinary albumin-to-creatinine ratio (UACR; OR=1.15 [1.10, 1.20]; *P*=2.28×10 ^10^), which was reported previously^70^, plus urinary sodium (OR=0.89 [0.86, 0.93]; *P*=8.80×10 ^8^) and urinary potassium (OR=0.91 [0.87, 0.95]; *P*=6.46×10 ^6^).

#### PheWAS on FECD index SNPs

We performed phenome-wide association scans (PheWAS) on the index variant of each locus in the FECD meta-analysis (Supplementary Table 6). In up to 458,296 MVP EUR participants, a total of 1,460 phenotypes were tested for each SNP: 1,170 phecodes^71^, 64 laboratory and vital signs measurements, and 225 survey questions. We found 31 associations with non-cornea traits that were significant after correction for the total number of tests (*P*<0.05/17,520). Among the significant pleiotropic associations of FECD risk alleles are a protective association with open-angle glaucoma at *SSBP3,* risk-increasing associations with benign colon neoplasms at laminin genes *LAMA5* and *LAMC1,* and an association with increased heart rate at *LAMB1,* which is replicated in the UK Biobank (*P*=2.0×10 ^8^)^72^.

#### Pleiotropy and co-localization analyses of TCF4 demonstrate link to renal function

The most significant PheWAS associations were observed at the *TCF4* risk allele, which was strongly associated with laboratory measurements of increased serum bicarbonate (*P*=7.0×10 ^62^), decreased chloride (*P*=9.1×10 ^24^), and increased potassium (*P*=2.3×10 ^9^) (Supplementary Fig. 6). This pleiotropy at *TCF4* likely explains the robust association with increased serum bicarbonate we previously observed in a phenome-wide comorbidity scan of FECD case-control status^19^.

Upon further evaluation of the *TCF4* locus in these significant laboratory measurement traits, we found that the index SNP of each trait (rs11659764) was the same as in FECD, and each displayed a highly similar complex pattern of local associations (Supplementary Fig. 7), which in FECD are thought to be caused by the partial LD of SNPs on different haplotypes with the CTG18.1 TNR. Validating our results, the same pattern was observed with externally derived UACR summary statistics^70^. We further found that the regression coefficients of significant SNPs at the *TCF4* locus were highly correlated across FECD and each of the four renal traits, suggesting co-localization. Positive correlation with FECD was observed in effect direction and magnitude for bicarbonate (*r*=0.91), potassium (*r*=0.77), and UACR (*r*=0.95), while negative correlation was observed with chloride (*r*=−0.90) (Supplementary Fig. 7).

In an effort to further untangle pleiotropic effects, we performed Bayesian colocalization analyses under the assumption of a single causal variant (the untyped CTG18.1 expansion) using coloc^73^. All four traits showed evidence of colocalization, with posterior probabilities >0.999 (Supplementary Table 7). We consider these observations strong evidence that the CTG18.1 expansion has pleiotropic effects on renal function. Moreover, the strength of the association with serum bicarbonate suggests that the effect of the CTG18.1 expansion on FECD may be mediated through dysregulation of ion transport in CECs.

**Figure 4:**
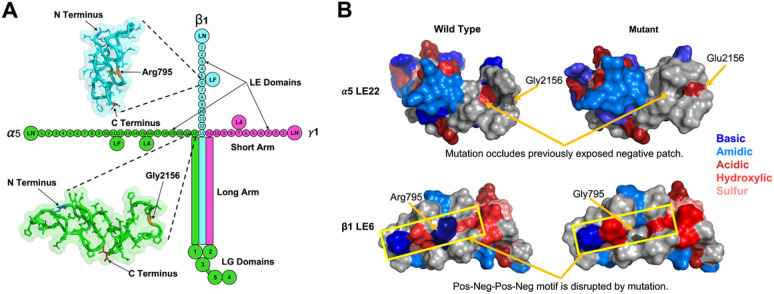
Structure of LM511 and predicted impact of missense variants in laminin genes *LAMA5* (α5) and *LAMB1* (β1). (a) Structural organization of the laminin-511 (LM511) heterotrimer. The green color denotes the LAMA5 subunit, blue denotes LAMB1, and pink denotes LAMC1. Significant FECD variants are located on the short arms of α5 and β1, in LE (laminin-type epidermal growth factor (EGF) like) domains LE22 and LE6, respectively. The insets depict AlphaFold 2 predictions of these domains, and the locations of the residues are shown in orange. (b) Top: Predicted surface structure of the α5 LE22 domain with and without the Gly2156Glu variant. Bottom: Predicted surface structure of the β1 LE22 domain with and without the Arg795Gly variant.

The gene products of two novel FECD loci at *LAMA5* and *LAMB1*, plus the known locus at *LAMC1* which we replicated, are the three subunits of the LM511 heterotrimer. Each monomer ( 5, β1, and γ1) of LM511 is a multi-domain polypeptide; these interact with each other to form the long arm of a cross-shaped structure, while their non-interacting portions constitute three short arms (Fig. 4A). The short arms are composed of laminin-type EGF-like (LE) domain repeats that terminate in a laminin N-terminal (LN) domain^74^. These short arms interact with other extracellular proteins to assemble and stabilize the BM, while the long arms facilitate interaction with cell surface receptors via globular domains.

The missense mutations at rs141208202 (*LAMA5*) and rs150990106 (*LAMB1*), correspond to a glycine to glutamic acid substitution at position 2156 of 5 LE22, and an arginine to glycine substitution at position 795 of β1 LE6, respectively. We examined the potential impact of these mutations on the structure and function of LM511, using SWISS-MODEL^75^ and AlphaFold 2 (AF2)^76^ to model the 5 LE22 and β1 LE6 domains (Supplementary Fig. 8A).

The glycine to glutamic acid substitution in 5 LE22 replaces a small hydrophobic residue with a large acidic one, altering the surface hydrophobicity and topology (Fig. 4B, top). The required orientation of 5 LE22, with respect to the cross, positions the mutated residue in close proximity to the other chains. This substantial change in LE22 may disrupt inter-chain interactions, and can also potentially destabilize the triple-helix of the long arm, which leads to disrupted interactions with cell surfaces through allosteric modulation of the LG domains.

Replacing the large basic arginine in β1 LE6 with a smaller hydrophobic glycine induces similar changes in surface hydrophobicity and topology (Fig. 4B, bottom). The wild-type arginine is part of a positive-negative-positive-negative patch on the LE6 domain surface that may present a binding motif. Breaking this motif can disrupt interactions to neighboring β1 domains or to other extracellular matrix proteins, resulting in binding affinity differences to the basement membrane and altered cell signaling.

While these two mutations have high potential to disrupt inter-domain interactions, it is unlikely that they will induce significant changes to the tertiary structures of 5 LE22 and β1 LE6. This is because LE domain backbones are covalently linked through four disulfide bonds that prevent any significant deviations from the native fold, resulting in no change in intra-hydrogen bond count for 5 LE22, and a loss of only three hydrogen bonds in β1 LE6 (Supplementary Fig. 8B). Correspondingly, Duet^77^ predicted that the β1 LE6 mutation is more destabilizing than the 5 LE22 mutation. Overall, our structural analysis suggests that the variants associated with FECD may destabilize LM511 through altered inter-domain interactions, rather than through structural changes of the mutated domains.

## Discussion

In this study we have identified eight novel genomic risk loci for FECD, and replicated the four existing loci, in the largest GWAS of FECD cases to-date (N_cases_=3,970). Our multi-ancestry analysis confirmed the considerably large effect of the *TCF4* locus across AFR and HIS ancestries; *TCF4* was the exclusive signal reaching GWS in these ancestry groups with smaller case numbers. Our results increase confidence in known FECD mechanisms, and our novel candidate genes expand our understanding of the role of laminins, collagen, integrins, and endothelial cell regulation in FECD pathophysiology and the maintenance of corneal deturgescence.

All three genes encoding subunits of LM511 had GWS associations with FECD in this study. Previously, LM511 has been primarily associated with tumor growth, both *in vitro* and *in vivo,* typically in the context of integrin-mediated adherence to tumor cells^78^. In a recent US cohort study where 68% of FECD cases were female, there were significantly higher risks in FECD cases for comorbidities with breast, thyroid, ovarian, and basal cell carcinomas^79^. Our PheWAS results also indicate the index variants at *LAMC1* and *LAMA5* are significantly associated with colon cancer (Supplementary Table 6). These findings suggest a potential link between LM511 and the increased risk of certain cancers observed in FECD cases. Another novel FECD gene encodes CD151, which modulates binding between laminins and integrins; CD151 overexpression is a marker of poor breast cancer survival and may also contribute to LM511 tumor response^78^.

Collagens are major components of the BM and Descemet’s membrane, and the “infiltration” of collagenous secretion (guttae) from Descemet’s membrane is a hallmark of FECD. Our GWAS results have associated *SSBP3*, which regulates COL1A2 (type I collagen), and *COL18A1* (type XVIII collagen) with FECD for the first time. Type XVIII collagen, which has been previously associated with Knobloch syndrome^49^, is found in BMs of nearly every tissue as well as the stromal side of Descemet’s membrane. Type XVIII collagen is also a HS proteoglycan; the product of another novel gene *HS3ST3B1* is responsible for generating binding sites for proteins on HS chains. FECD CEC samples have been previously shown to contain higher levels of keratan sulfate, a sulfated glycosaminoglycan (GAG) found in the ECM^58^, and our results suggest HS-GAGs may have a similar (lubricating) role in FECD.

Our findings corroborate previous reports of correlation between corneal and renal traits. We found that the PGSs for the renal trait UACR, which has been associated with several retinal disorders, along with urinary sodium and urinary potassium, had highly significant associations with FECD, secondary only to other corneal traits (Supplementary Table 5). The *TCF4* locus has also been associated at GWS with these renal measures in GWAS Catalog. Similarly, previous experimental analysis of corneal endothelium in samples of FECD with the CTG18.1 TNR expansion found increased expression of genes involved in ion transport^80^. Our analysis demonstrated for the first time a high probability of colocalized causal structure between FECD and renal-associated ionic measurements (Supplementary Fig. 7). This included a novel relationship of FECD with serum bicarbonate, which has been previously shown to mediate lactate-coupled water flux in the corneal endothelial pump^81^.

Furthermore, the novel FECD gene products of LM511 and COL181A play a role in FECD as well as kidney disease, and this may be due to concurrent embryonic patterning in renal and corneal endothelial cells; the kidney and eye develop around the same time during organogenesis around the fourth through sixth gestational weeks^82^. LM511 and *COL181A* are both highly expressed in tubule BMs. Polycystic kidney disease (PKD) cells have reduced LM511 and overexpressed LM332, and a mutation causing loss of *LAMA5* function induced PKD in a mouse model^83^. The *LAMC1* locus has been previously associated in GWAS with several renal traits, including estimated glomerular filtration rate, type 2 diabetes, diastolic blood pressure, and red blood cell count. This study also replicated a known FECD risk locus at *ATP1B1,* which is highly expressed in CECs, and whose gene product regulates sodium balance as a subunit of Na^+^/K^+^ ATPase.

Our analysis contains several limitations. First, the algorithm we used to identify FECD cases^19^, while clinically validated, was based solely on electronic health record diagnoses, and not the slit lamp imaging used previously^26^, which may have diluted the phenotyping in our analysis. We were constrained by the demographics of FECD cases in the MVP dataset; FECD is more common in women, but our sample, and MVP in general, skew heavily male, which may have biased our GWAS towards the identification of male-specific genetic factors. We also did not differentiate between rare early-onset and more common late-onset FECD, whose pathophysiologies may involve separate genetic mechanisms^25^.

It is well established that the most predictive FECD allele at 18q21.2 is the CTG18.1 TNR expansion^84^. Our GWAS used chip-based genotyping, so we relied on SNPs tagging CTG18.1 alleles instead of direct genotyping. Although our lead *TCF4* SNP rs11659764 is an imperfect proxy for CTG18.1, it nonetheless showed a strong and consistent association signal across multiple ancestry groups.

AlphaFold 2 and SWISS-MODEL are accurate in single-state predictions, but a limitation arises as they provide no information on protein fluctuations, leading to lower confidence in the structure of intrinsically disordered regions (IDR). While crystal structures of homologs suggest that there are no significant IDRs in LM511 LE domains, the question of how mutations can affect their dynamics remains. Although the predicted single-state structures used here do not capture shifts in dynamics, they nonetheless inform that the mutations significantly change surface chemistry and topology, and by extension, interactions to binding partners. Additionally, AlphaFold 2 is trained on wild-type protein structures and therefore has limited ability to predict when missense mutations will cause changes in protein folding^85^; however, the backbone structure of LM511 indicates folding changes are not likely to occur from as a result of our FECD risk alleles, and so this limitation should not impact our functional predictions.

Our GWAS results have added eight novel genomic risk loci to the previous total of sixteen genes known to be associated with FECD^84^. We were able to place these novel loci into biological contexts that are compatible with currently understood mechanisms of FECD disease progression. Additionally, the MVP dataset enabled unprecedented quantitative analyses of non-EUR cohorts^32^, and this analysis expands our understanding of the shared genetic architecture of FECD in these populations. We hope these results can lead to improved genetic risk prediction, and once experimentally validated, will help inform modern treatment strategies.

## Methods

### Ethics/study approval.

Informed consent was obtained from all participants, and all studies were performed with approval from the Institutional Review Boards at participating centers.

### Phenotyping.

We previously developed a FECD case-control algorithm based on structured electronic health record data and confirmed its accuracy by chart review at three VA Medical Centers^19^. We applied the algorithm to conduct genome-wide association analyses (GWAS) and to analyze associated electronic health record (EHR) data.

### QC and imputation.

MVP samples were genotyped on the Thermo Fisher MVP 1.0 Axiom array. The design and QC of the array is described in detail elsewhere^86^. Genotypes were phased using SHAPEIT4^87^ and imputed to the TOPMed reference panel (version R2) using Minimac4.

### GWAS.

Samples were classified according to genetic ancestry using the HARE (Harmonized Ancestry and Race/Ethnicity) method^88^. GWAS analyses were performed on ancestry-stratified subsets in MVP using SAIGE v1.1.6.2^33^, adjusting for sex, age, mean-centered age^2^, and 10 ancestry-specific principal components. To ensure accurate effect size estimation, Firth approximation was applied to SNPs with *P* < 0.05. Association scans were performed on well-imputed SNPs (INFO > 0.5) using an ancestry-specific minor allele frequency (MAF) cutoff of ≥ 0.1%.

### GWAS meta-analysis.

We performed inverse variance-weighted fixed effects meta-analyses of GWAS summary statistics. First, we performed a EUR GWAS meta-analysis of MVP EUR and Afshari et al.^26^ summary statistics, followed by a multi-ancestry GWAS meta-analysis of MVP EUR, Afshari et al.^26^, and MVP AFR summary statistics. (MVP HIS was excluded from the multi-ancestry meta-analysis due to containing <100 cases.) Each set of summary statistics was converted into GWAS-VCFs using the +munge plug-in (https://github.com/freeseek/score) of bcftools v1.16^89^. The Afshari et al.^26^ summary statistics were lifted over to the GRCh38 genome build using the +liftover plug-in. Finally, Meta-analyses were performed using the +metal plug-in. For the multi-ancestry meta-analysis, a cohort-specific MAF cutoff ≥ 1% was applied.

### Characterizing significant loci.

We used the COJO-slct method implemented in GCTA^90^ version 1.94.1 to find conditionally independent genome-wide significant secondary signals at significant loci. An LD reference panel was constructed from 100,000 randomly selected MVP EUR subjects. The *TCF4* locus was excluded from COJO analysis due to the association of the untyped CTG18.1 repeat expansion. Variants for each independent genomic risk locus in the multi-ancestry meta-analysis were clumped and lead variants were identified using the Functional Mapping and Annotation of Genome-Wide Association Studies (FUMA) web server (v1.4.2)^91^. The maximum *P*-value cutoff was set to 0.05, and a first LD threshold of *r*^*2*^ ≥ 0.6 and second threshold of *r*^*2*^ ≥ 0.1 were used to define loci and lead SNPs. The maximum distance between LD blocks to merge loci was 250 Kb. Pleiotropy of significant loci with previous GWAS traits was identified using GWAS Catalog via FUMA, and LDtrait^92^, using a 250 Kb range.

### LDSC.

Non-partitioned liability score heritability for FECD and pairwise genetic correlations (*r*_*g*_) between FECD and ocular traits were computed using Linkage Disequilibrium score regression (LDSC) v1.0.1^35^. Summary statistics for keratoconus^47^, CCT^65,66^, and CRF^67^, were obtained from GWAS Catalog; corneal hysteresis (CH) was provided by the authors^68^. Prior to computing *r*_*g*_, all summary statistics were quality controlled and alleles were harmonized to the reference genome using MungeSumstats v1.7.8^93^.

### SuSiE fine-mapping.

Genome-wide significant loci in the EUR meta-analysis were fine-mapped using SuSiE^39,40^ version 0.11.42. Pairwise SNP LD matrices were constructed from imputed dosages over the same sample set used in the MVP EUR GWAS (N=254,596) using LDSTORE 2.0. Default options were used, including the maximum number of causal variants at a locus (10). The *TCF4* locus was excluded from this analysis due to the association of the untyped CTG18.1 repeat expansion.

### Associations of PGSs with FECD.

Phenome-wide polygenic score files were obtained from European Molecular Biology Laboratory’s European Bioinformatics Institute (EMBL-EBI) PGS Catalog^69^. All European-ancestry subjects in MVP were scored across all available PGSs using the +score plugin (https://github.com/freeseek/score) of bcftools v1.16^89^. PGSs were then loaded into the dosage format field of VCFs readable by SAIGE for association testing. To determine pleiotropy of genetic predisposition to traits on FECD, logistic regression was used to examine associations of PGSs on MVP EUR FECD cases and controls using SAIGE v1.1.6.2^33^, adjusting for the same covariates as in GWAS (sex, age, mean-centered age^2^, and 10 ancestry-specific principal components).

### PheWAS of index SNPs.

We performed a PheWAS on each individual index SNP using summary statistics generated from the August 2022 beta release of the genome-wide PheWAS (gwPheWAS) project in MVP. Genotypes were imputed using the African Genome Resource and 1000 Genomes imputation panels. Phenotypes were derived from phecodes following standard definitions^71^, a baseline survey distributed to all MVP enrollees, as well as EHR-based laboratory and vital signs measurements. A GWAS was performed on each SAIGE using sex, age, age-squared, and 10 principal components as covariates.

### Colocalization.

Genetic associations in MVP EUR participants at the *TCF4* locus (chr18:50,000,000-60,000,000) for serum bicarbonate, chloride, and potassium were obtained using PLINK 2.0 alpha 4^94^. Traits were rank-based inverse normal transformed (RINT), and linear regression was performed using sex, age, age-squared, and 20 principal components. Genotype QC was performed as in the FECD GWAS described above. RINT urinary albumin-to-creatinine ratio (UACR) summary statistics^70^ for EUR were obtained from GWAS Catalog, and lifted from hg19 to hg38. Effect comparison plots include only variants from chr18:54,500,000-56,500,000 with *P*<0.001; effect correlation was measured using Pearson’s *r.* Single causal variant colocalization was performed on summary statistics using the colic.abf() function in Coloc v5.2.1^95^. A posterior probability > 0.9 for Hypothesis 4 (both traits are associated and share a single causal variant) was used as the criteria for colocalization.

### Structural analysis.

Because no known crystal structures of human 5 LE22 and β1 LE6 exist, we modeled them using two structure prediction tools. SWISS-MODEL^75^ was used to homology model the domains; the template with the highest GMQE score was selected. AI-based AlphaFold 2^76^ (AF2) was used to supplement SWISS-MODEL for the missing portions in the homology-based template (Supplementary Fig. 8A). Structural differences between the SWISS-MODEL and the rat homolog, and those between SWISS-MODEL and AF2 predictions were both within the range of thermal fluctuations, lending confidence to the AF2 predictions. DUET was used to predict the change in protein stability due to the mutations^77^.

## Figures and Tables

**Table 1.** Association of the top SNP at the *TCF4* locus, rs11659764, in MVP cohorts. In European (EUR), admixed African (AFR), and Hispanic/Latino (HIS) cohorts, rs11659764 had the most significant association with FECD. The minor allele increased odds ratio of FECD risk consistently across ancestry groups despite differences in effect allele frequency (EAF). CI confidence interval.

Ancestry	Odds ratio [95% CI]	*P*-value	EAF cases	EAF controls
EUR	6.41 [5.86, 7.01]	9.4×10 ^360^	0.222	0.045
AFR	7.57 [4.87, 11.75]	1.1×10 ^19^	0.061	0.009
HIS	7.16 [3.93, 13.04]	6.2×10 ^11^	0.131	0.022

**Table 2.** Genome-wide significant loci in the multi-ancestry meta-analysis of FECD. Genomic risk loci from the meta-analysis of MVP European and African cohorts plus Afshari et al.^26^. We identified eight novel FECD loci, and replicated all four previously reported loci. rs1138714 previously reached suggestive significance in Afshari et al.^26^ at *P*=7x10^−7^. Genomic coordinates correspond to GRCh38. EA, effect allele; NEA, non-effect allele; EAF, effect allele frequency; SE, standard error; OR [L95, U95], odds ratio with lower and upper bounds of 95% confidence interval; Direction, SNP effect direction in MVP EUR, MVP AFR, and Afshari et al.^26^ meta-analysis cohorts, respectively. “?“ indicates the AFR variant did not meet the allele frequency cutoff of 1% and was not included.

rsID	Chr:Pos	Predicted causal gene	EA/ NEA	EAF	N case	N	OR [L95, U95]	*P*- value	Direction
**Novel loci (*P* < 5 × 10 ^8^)**									
rs11590557	1:54,324,099	*SSBP3*	A/G	0.04	3,655	258,564	1.61 [1.43,1.81]	6.86 × 10^−15^	+?+
rs74882680	7:11,700,254	*THSD7A*	G/A	0.02	3,655	258,564	1.72 [1.48, 2.00]	2.78 × 10^−12^	+?+
rs150990106	7:107,955,927	*LAMB1*	A/G	0.02	3,655	258,564	1.75 [1.45, 2.10]	4.33 × 10^−9^	+?+
rs1138714	11:825,110	*CD151, PIDD1*	G/A	0.53	3,970	337,764	1.22 [1.16, 1.28]	3.01 × 10^−14^	+++
rs12439253	15:60,764,393	*RORA*	T/G	0.08	3,970	337,764	1.29 [1.18, 1.40]	4.31 × 10^−9^	+−+
rs9303111	17:14,663,407	*HS3ST3B1*	C/A	0.32	3,970	337,764	0.81 [0.76, 0.85]	1.17 × 10^−13^	−−−
rs141208202	20:62,322,048	*LAMA5*	T/C	0.05	3,655	258,564	1.40 [1.25, 1.57]	1.42 × 10^−8^	+?+
rs114065856	21:45,432,844	*COL18A1*	T/C	0.04	3,970	337,764	0.61 [0.52, 0.72]	2.87 × 10^−9^	−−−
**Previously reported loci**									
rs79742895	1:62,317,189	*KANK4*	C/T	0.04	3,655	258,564	1.78 [1.59, 1.98]	1.78 × 10^−24^	+?+
rs1200114	1:169,091,251	*ATP1B1*	A/G	0.66	3,970	337,764	0.73 [0.69, 0.77]	5.38 × 10^−34^	−−−
rs2093985	1:183,125,187	*LAMC1*	T/C	0.54	3,970	337,764	0.80 [0.76, 0.84]	2.58 × 10^−18^	−−−
rs11659764	18:55,668,281	*TCF4*	A/T	0.05	3,655	258,564	7.15 [6.60, 7.74]	8.60 × 10^−509^	+?+

**Table 3.** Summary of novel candidate genes. Candidate genes for eight novel loci identified in the multi-ancestry meta-analysis for FECD.

Novel candidate gene	Putative function
*SSBP3*	Likely binds to polypyrimidine promoter of *COL1A2,* regulating transcription.
*THSD7A*	Regulation of endothelial cell migration and adhesion via binding of integrin αvβ3.
*LAMB1*	Beta-1 subunit of laminin-511, component of basal lamina.
*CDC151; PIDD1*	Global regulator of endothelial cell-cell and cell-matrix adhesion; may have a role in regulating corneal endothelial cell death via apoptosis.
*RORA*	Member of retinoic acid-related orphan receptors (RORs) family, which play a role in embryonic development of the eye.
*HS3ST3B1*	Regulates heparan sulfate, which may have a role in corneal homeostasis.
*LAMA5*	Alpha-5 subunit of laminin-511, component of basal lamina.
*COL18A1*	Collagen type XVIII subunit, is cleaved to form endothelial cell regulator endostatin.

## Data Availability

The full summary level association data from the meta-analysis and individual population association analyses in MVP will be available via the dbGaP study accession number phs001672.
